# The effect of phased written health education combined with healthy diet on the quality of life of patients after heart valve replacement

**DOI:** 10.1186/s13019-021-01437-7

**Published:** 2021-06-25

**Authors:** Dan Li, Pujuan Liu, Huijun Zhang, Li Wang

**Affiliations:** 1 Department of Cardiac Surgery, The First Hospital of Hebei Medical University, Shijiazhuang, China; 2 Functional Division, The Fourth Hospital of Hebei Medical University, No.169 Tianshan Street, Yuhua District, Shijiazhuang, 050000 Hebei China

**Keywords:** Phased written health education, Healthy diet, Heart valve replacement, Quality of life

## Abstract

**Background:**

To observe the effect of phased written health education combined with healthy diet on the quality of life of patients after heart valve replacement.

**Methods:**

One hundred-thirty patients who underwent heart valve replacement surgery in our hospital from January 2018 to January 2020 were enrolled as the research subjects. They were randomly divided into study group (65 patients, phased written health education combined with health Diet) and control group (65 cases, routine health education). The drug compliance and the degree of anticoagulant drugs knowledge were compared between the two groups in the first and second stage rehabilitation and the recovery stage. The health behavior ability and quality of life at different phases were also observed.

**Results:**

During the first and the second stage rehabilitation, and the recovery stage, the drug compliance of the study group was superior to that of the control group, with statistical significance (*P* < 0.05). Meanwhile, the knowledge of anticoagulant drugs in the study group was better than that in the control group, with statistical significance (*P* < 0.05). Before intervention, there was no significant difference in health behavior ability and quality of life between the two groups (*P* > 0.05). The healthy behavior ability of study group for each stage was superior to the control group, the difference was significant (*P* < 0.05). The healthy behavior ability and quality of life showed the same results with statistical significance (*P* < 0.05).

**Conclusion:**

The use of phased written health education combined with healthy diet in patients after heart valve replacement can effectively improve patients’ medication compliance, anticoagulant drugs knowledge, healthy behavior ability and quality of life at different stages, and is worthy of clinical application.

## Background

Valvular heart disease (VHD) is a common disease [1, 2] With the aging of China’s population increasing in recent years, the incidence of this disease is increasing year by year. The main clinical symptoms of VHD are dyspnea after a little exercise, or even paroxysmal dyspnea at night, and the inability to rest supine, which poses a serious threat to the patient’s physical and mental health. Heart valve replacement is often used to treat heart valvular disease and has achieved good results. However, patients should take anticoagulant drugs for a lifetime or for a long time after the operation. If the drugs are used improperly or not as required, bleeding or embolism will occur. Bleeding and embolization caused by improper use of drugs account for 75% of long-term postoperative complications, which not only have a serious impact on postoperative quality of life, but also are the leading cause of death [3] Therefore, how to effectively improve the treatment compliance of patients after cardiac valve replacement is one of the key topics of clinical research. Health education is often given to patients after heart valve replacement to enable them to understand anticoagulant drugs, but it has certain limitations and cannot improve patients’ treatment compliance, thus affecting the quality of life of patients. Phased written health education is designed for patients according to the characteristics of different stages after surgery, and combined with healthy diet to obtain the expected results [4–6] This study will explore the effect of a combination of phased written health education and healthy diet on the quality of life of patients after heart valve replacement.

## Methods

### Clinical data

A total of 130 patients who underwent cardiac valve replacement in our hospital from January 2018 to January 2020 were enrolled in our study. All enrolled patients were randomly divided into study group and control group with 65 cases each. The demographic characteristics of the patients in the study group are as follows: The age range is 28.63–74.38 years old, the average age is 53.83 ± 3.21 years old. The gender distribution was 39 males and 26 females. Mitral valve replacement was performed in 22 cases, aortic valve replacement in 23 cases, simultaneous aortic and mitral valve replacement in 20 cases. Concurrent surgeries included: 42 cases of mechanical valve replacement in, 22 cases of biological valve replacement, 7 cases of thromboclearance in the left atrium, 19 cases of tricuspid valve plasty, 5 cases of mitral valve plasty, 3 cases of mitral valve vegetations removal, 2 cases of coronary artery bypass grafting, 1 case of repair of superior mesenteric artery pseudoaneurysm, and 1 case of modified trunk stent implantation. The demographic characteristics of the patients in the control group are as follows: The age range is 28.59–75.72 years old, the average age is 53.61 ± 3.42 years old. The gender distribution was 38 males and 27 females. Mitral valve replacement was performed in 21 cases, aortic valve replacement in 23 cases, simultaneous aortic and mitral valve replacement in 21 cases. Concurrent surgeries included: 41 cases of mechanical valve replacement, 23 cases of biological valve replacement, 8 cases of thromboclearance in the left atrium, 18 cases of tricuspid valve plasty, 6 cases of mitral valve plasty, 6 cases of mitral valve vegetations removal, 2 cases of coronary artery bypass grafting, 1 case of repair of superior mesenteric artery pseudoaneurysm, and 1 case of modified trunk stent implantation. Baseline characteristics were comparable between the two groups(*P* > 0.05). The formulation of this research protocol complies with the relevant requirements of the Declaration of Helsinki of the World Medical Association.

### Inclusion and exclusion criteria

Inclusion criteria: (1) with complete clinical data; (2) with normal spirit and good compliance; (3) willing to participate; (4) provided of informed consent.

Exclusion criteria: (1) combined with serious organic diseases; (2) accompanied by serious cardiovascular or cerebrovascular diseases; (3) inability to communicate properly.

## Methods

The control group was given routine health education. The specific contents are as follows: (1) Informing the patients that they need to take the medication on time and in the amount, and recorded the medication. (2) Rechecking the international normalized ratio to determine if the dosage of anticoagulant was appropriate for the patients. (3) Paying attention to identify early clinical signs of bleeding or thrombosis due to excessive or insufficient use of anticoagulants. (4) Women of childbearing age were advised to use contraception and to consider pregnancy at least 2.5 years after surgery under the guidance of a doctor. (5) Preventing trauma as much as possible. (6) When receiving treatment or undergoing various traumatic examinations, it is necessary to inform the doctor to avoid bleeding events.

On the basis of the control group, the study group was given phased written health education combined with healthy diet. A phased written health education team was established, consisting of 1 assistant chief physician, 1 head nurse, 6 senior nurses, and 1 nutritionist. The staff must had worked in our hospital for more than 5 years. The team members had undergone a one-month training (December 2018), mainly studying the phased health education of heart valve replacement surgery. According to the characteristics of recovery after heart valve replacement, the nursing process was divided into 3 phases, namely the first-stage rehabilitation (from the day after the operation to the day of discharge), the second-stage rehabilitation (from the day of discharge to within 2 months after the operation) and third stage (recovery stage, from 2 months to 6 months postoperatively). According to the characteristics of the patients’ recovery at different stages, a health education and healthy diet manual was formulated and distributed to the patients to ensure that each person had one copy. The senior nurses in the team established a WeChat group. The patients and their relatives, as well as the nurses participating in this research, were added to the chat group to facilitate the communication between patients and nursing staff. The nursing staff should answer the questions raised by the patients. At the same time, a WeChat public account was established, and at 8 o’clock in the morning every Monday and Wednesday, the content of the precautions after heart valve replacement was pushed on time to guide patients to learn relevant knowledge. Primary nurses gave health education to patients at 3 pm every Friday, about 45 min each time, once a week. During the intervention period, a total of 24 health education was conducted, and the content of health education was released through the WeChat public account. At the same time, patients were organized to communicate in the WeChat group twice a week, each time for 40–50 min. A week before each health education, the primary nurses needed to inform the patients and their relatives of the specific time for health education by phone or by WeChat, and reminded the patients to check the content of WeChat. Once the health education was released, the patients should be told to read it as soon as possible. After that, the primary nurses should organize the patients to communicate in the WeChat group. Team members needed to answer patients’ questions in detail. In addition, patients could communicate with each other and share their experience of rehabilitation.

**Fig. 1:**
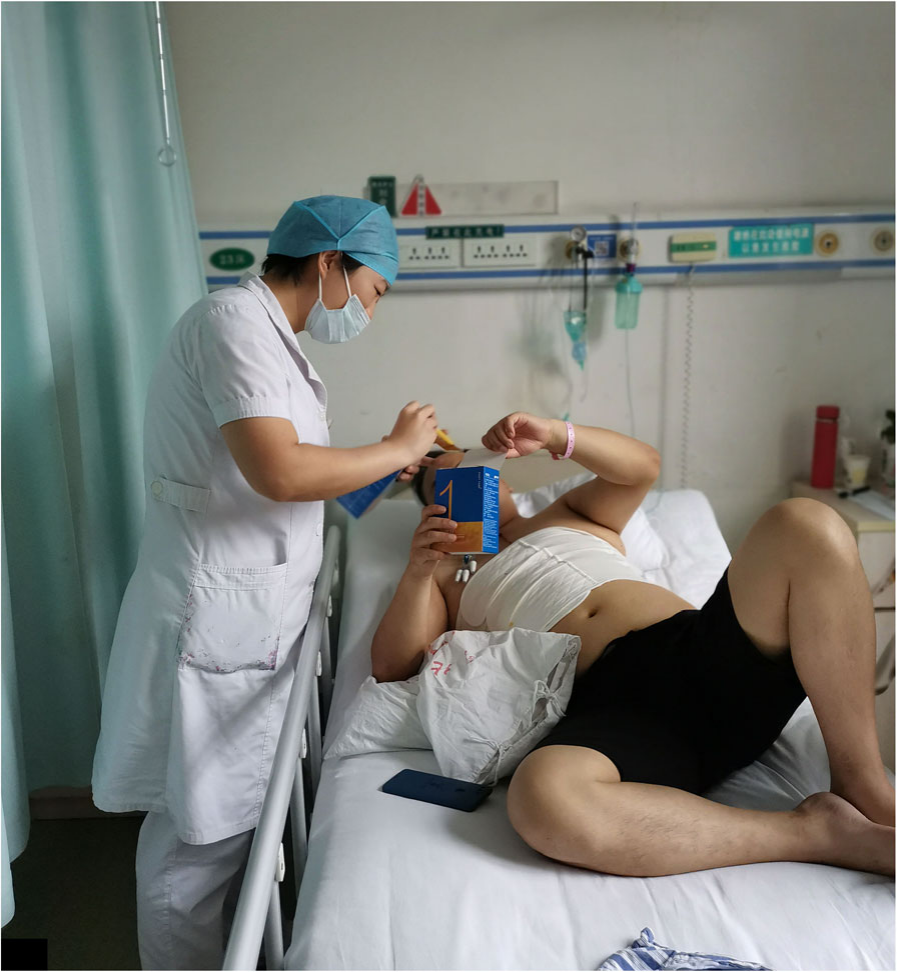
The nursing staff informed the patients in detail about the use of the anticoagulant warfarin and the matters needing attention

The content of the phased health education are as follows: (i) First stage rehabilitation: Anticoagulant therapy was given within 24 h after operation, and the anticoagulant drug warfarin was taken orally at dose. Meanwhile, the patients’ thrombin time was measured regularly, and the warfarin dose was adjusted timely until the thrombin time reached the normal level. The patients and their relatives were informed of the dosage and method of the drug used in detail, and the relatives should participate in the whole process of taking medicine. Meanwhile, visual metaphors and repeated explanations was used to explain the methods of taking medicine (see Fig. 1), so that patients could fully understand anticoagulant drugs and other related knowledge, possible complications and countermeasures. Informed the patients of the importance of regular testing, so that each patient can correctly grasp the time of drug use, dosage, possible complications and precautions, etc. Praised the behavior of taking medication actively to improve patients’ enthusiasm and cooperation in taking medication. When the patients came around from the anaesthetic and the vital signs were stable, auscultated the patient’s breath sounds, guided the patient to perform abdominal breathing and deep breathing, and assisted the patients in passive movement of the limbs. Patients could try bedside activities 1–3 days after surgery, such as standing alone by the bed or sitting on a chair. The primary nurses should actively communicate with the patients, grasped the patients’ psychological characteristics, and conducted psychological counseling. The primary nurses should tell the patients and their relatives to eat easy-to-digest, bland foods, mainly low-salt, low-fat, and high-fiber foods. Patients should be advised to avoid eating foods like pig liver, spinach, carrots, etc. These foods are rich in vitamin K, which can antagonize anticoagulant drugs and shorten prothrombin time. (ii) Second stage rehabilitation: Instructed the patients to continue to take medication as prescribed, and reminded the patients to take medication daily through WeChat. The patients’ heart function (cardiopulmonary exercise test and 6-min walking test) was evaluated every week before participating in health education. The tests should be terminated, if the patients developed cyanosis, dizziness, blood pressure reduction, the patients actively requested to stop the tests, or electrocardiogram showed pathological Q waves during the tests. The patients’ extreme exercise volume and the second-largest exercise volume were evaluated using the maximum extreme exercise state of cardiopulmonary exercise and the distance of 6-min walking test. The nursing staff guided patients to conduct self-care including guidance on life behaviors, and consulted relevant materials. After sorting out the materials that were helpful to the patients’ recovery, sent them to the patients. Formulated exercise programs for patients such as physical resistance, Tai Chi, yoga, balance, etc. Reminded patients to pay attention to healthy diet, avoid drinking beverages and foods containing additives, carbonated beverages, quit smoking and alcohol, pay attention to body weight. Once the patients’ weight exceeded the normal weight, they should control their diet to keep weight. (iii) Recovery stage: Instructed patients to live a healthy life and consolidate self-care behaviors. According to patients’ questions, nurses constantly supplement health education and dietary guidance.

### Observation index

The knowledge and compliance of anticoagulant drugs in the first and second stage of rehabilitation, and recovery stage of the two groups were compared. The healthy behavior ability and quality of life in each stage of both groups were observed.

#### Medication compliance

The medication compliance of all patients at all stages was evaluated by the Modified Morisky Scale (MMS) [7] There are 4 questions in this scale, with 3 points for each question. The higher the score, the worse the compliance. Complete compliance: ≤ 4 points. Incomplete compliance: 5–8 points. Complete noncompliance: ≥ 9 points.

#### Degree of anticoagulant drugs knowledge

A questionnaire designed by our hospital was used to evaluate the degree of anticoagulant drugs knowledge in patients, a total of 8 items in 4 aspects, including risk factors and importance of anticoagulant drugs with anticoagulant knowledge. Mastery: Correctly answered more than 7 items. Partial mastery: Correctly answer 5 or 6 items. Failure to master: Correctly answered less than or equal to 4 items. Total mastery rate = partial mastery rate + mastery rate.

#### Healthy behavior ability

All patients were evaluated using the Healthy Behavior Ability Scale, [8] which has 28 items from the 4 dimensions of psychological well-being, health responsibility, nutrition management, and exercise management, and uses Likert scale, the total score is 0–112 points. The higher the score, the higher the patients’ healthy behavior ability.

#### Quality of life

The quality of life of all patients was assessed using the World Health Organization Quality of Life (WHOQOL-BREF) [9] The scale has 5 areas including environment, social relations, independence, psychology and physical health. There are 26 items in total and the score is 1–5 points, the total score is 0–130 points. The higher the score, the higher the patient’s quality of life.

### Statistical analysis

Data were statistically analyzed using statistical software SPSS21.0. Measurement data were expressed as mean ± standard deviation ($\overline{x}$ ± SD) and evaluated using a t-test. Count data were expressed as a percentage and compared using a Chi-square test. *P* < 0.05 was considered statistically significant.

## Results

### Comparison of medication compliance between the two groups

Cardiac valve replacement succeed in all patients. In each period, the complete compliance rate of the study group was higher than that of the control group, with significant differences (*P* < 0.05), were shown in Table 1.

**Table 1:** Comparison of medication compliance between two groups

Period	Study group(*n* = 65)	Control group(*n* = 65)	χ^2^	*p* value
First stage of rehabilitation				
Complete compliance	64 (98.46)	53 (81.54)	10.342	0.001
Incomplete compliance	1 (1.54)	9 (13.85)	7.073	0.008
Complete noncompliance	0	1 (1.54)	1.008	0.315
Second stage of rehabilitation				
Complete compliance	63 (96.92)	52 (80.00)	9.119	0.003
Incomplete compliance	2 (3.08)	11 (16.92)	6.766	0.009
Complete noncompliance	0	2 (3.08)	2.031	0.154
Recovery stage				
Complete compliance	62 (95.38)	51 (78.46)	8.188	0.004
Incomplete compliance	2 (3.08)	12 (18.46)	8.005	0.009
Complete noncompliance	1 (1.54)	2 (3.08)	0.341	0.559

*n*(%)

### Comparison of degree of anticoagulant drugs knowledge between the two groups

In each stage, the degree of anticoagulant drug knowledge of the study group was superior to that of the control group, with significant difference (*P* > 0.05), were shown in Table 2.

**Table 2:** Comparison of the degree of anticoagulant drugs knowledge between the two groups

Period	Study group (*n* = 65)	Control group(*n* = 65)	χ^2^	*p* value
First stage of rehabilitation				
Mastery	35 (53.85)	23 (35.38)	4.483	0.034
Partial mastery	29 (44.62)	34 (52.31)		
Failure to master	1 (1.54)	8 (12.31)		
Total mastery rate	64 (98.46)	57 (87.69)	5.849	0.016
Second stage of rehabilitation				
Mastery	34 (52.31)	20 (30.77)	6.209	0.013
Partial mastery	29 (44.62)	32 (49.23)		
Failure to master	2 (3.08)	13 (20.00)		
Total mastery rate	63 (96.92)	52 (80.00)	9.119	0.003
Recovery stage				
Mastery	33 (50.77)	21 (32.31)	4.561	0.033
Partial mastery	29 (44.62)	31 (47.69)		
Failure to master	3 (4.62)	2 (3.08)		
Total mastery rate	62 (95.38)	13 (20.00)	8.188	0.004

*n* (%)

### Comparison of health behavior ability between the two groups

Before intervention, there was no significant difference in health behavior ability between the two groups (*P* > 0.05). The healthy behavior ability of the study group at all stages was better than that of the control group, with significant difference (*P* < 0.05), were shown in Table 3.

**Table 3:** Comparison of healthy behavior ability of the two groups ($\overline{x}$ ±s)

Period	Study group(n = 65)	Control group(*n* = 65)	*p* value
Pre-intervention stage			
Nutrition management	15.02 ± 1.76	14.98 ± 1.67	0.894
Responsibility for health	16.12 ± 1.43	16.09 ± 1.51	0.908
Sport Management	13.01 ± 0.27	13.08 ± 0.29	0.157
Psychological well-being	14.56 ± 0.76	14.62 ± 0.65	0.629
Total points	58.80 ± 4.22	58.77 ± 4.12	0.967
First stage of rehabilitation			
Nutrition management	18.61 ± 2.91	16.69 ± 2.76	<0.001
Responsibility for health	20.37 ± 3.01	18.41 ± 3.27	0.001
Sport Management	16.93 ± 2.14	14.89 ± 2.31	<0.001
Psychological well-being	18.38 ± 1.87	16.41 ± 1.92	<0.001
Total points	55.91 ± 9.93	66.40 ± 10.26	<0.001
Second stage of rehabilitation			
Nutrition management	24.29 ± 2.87	22.19 ± 2.39	<0.001
Responsibility for health	24.91 ± 3.14	22.56 ± 3.23	<0.001
Sport Management	25.39 ± 2.23	22.01 ± 2.19	<0.001
Psychological well-being	24.98 ± 2.14	21.01 ± 2.31	<0.001
Total points	99.57 ± 10.38	90.08 ± 10.12	<0.001
Recovery stage			
Nutrition management	24.87 ± 2.95	22.65 ± 2.53	<0.001
Responsibility for health	25.15 ± 3.28	22.78 ± 3.98	<0.001
Sport Management	25.65 ± 2.56	22.22 ± 2.31	<0.001
Psychological well-being	25.28 ± 2.23	21.26 ± 2.65	<0.001
Total points	100.95 ± 11.02	88.91 ± 11.47	<0.001

### Comparison of quality of life between the two groups

Before intervention, there was no significant difference in quality of life between the two groups (*P* > 0.05). The quality of life of the study group at all stages was better than that of the control group, with significant differences (*P* < 0.05), were shown in Table 4.

**Table 4:** Comparison of quality of life of the two groups ($\overline{x}$ ±s)

Period	Study group(*n* = 65)	Control group(*n* = 65)	*p* value
Pre-intervention stage			
Environment	19.28 ± 3.37	19.31 ± 3.02	0.958
Social relations	19.63 ± 2.28	19.67 ± 2.31	0.921
Independence	19.03 ± 3.21	19.11 ± 3.19	0.887
Physical health	18.74 ± 1.93	18.81 ± 2.01	0.839
Physiological	4.98 ± 0.39	4.91 ± 0.34	0.277
Total points	81.66 ± 11.18	81.81 ± 10.87	0.938
First stage of rehabilitation			
Environment	25.28 ± 3.41	22.31 ± 3.45	<0.001
Social relations	25.47 ± 2.34	22.08 ± 2.76	<0.001
Independence	26.43 ± 3.23	22.21 ± 2.83	<0.001
Physical health	31.03 ± 2.38	24.39 ± 2.42	<0.001
Physiological	9.01 ± 1.02	6.05 ± 1.01	<0.001
Total points	117.22 ± 12.38	97.04 ± 12.47	0.018
Second stage of rehabilitation			
Environment	26.76 ± 3.52	22.65 ± 3.53	<0.001
Social relations	26.61 ± 3.01	23.21 ± 2.87	<0.001
Independence	26.54 ± 3.19	22.36 ± 2.91	<0.001
Physical health	32.78 ± 2.46	24.87 ± 3.01	<0.001
Physiological	9.08 ± 0.78	6.11 ± 1.23	<0.001
Total points	121.77 ± 12.96	99.20 ± 13.55	<0.001
Recovery stage			
Environment	26.83 ± 3.61	22.79 ± 3.59	<0.001
Social relations	26.75 ± 2.98	23.16 ± 2.91	<0.001
Independence	26.63 ± 3.25	22.47 ± 2.94	<0.001
Physical health	33.26 ± 2.87	24.93 ± 2.48	<0.001
Physiological	9.18 ± 0.68	6.45 ± 1.31	<0.001
Total points	122.65 ± 13.39	99.80 ± 13.23	<0.001

## Discussion

### Effect of phased written health education combined with healthy diet on medication compliance of patients after heart valve replacement

Valvular heart disease is often treated with heart valve replacement, while heart valve replacement usually uses artificial mechanical valves or artificial biological valves for replacement. Valve replacement is a foreign body to the heart. If the valve comes in contact with blood, it is easy to form thrombus and affect valve function. Therefore, patients should be treated with anticoagulant drugs for a long time or for a lifetime after heart valve replacement. Many studies have suggested that patients after heart valve replacement were prone to fail to follow the doctor’s advice and take medicine in a timely manner after discharge due to lack of knowledge about the disease. The longer the time after discharge, the worse the compliance. Then it will cause many anticoagulant complications, which will seriously affect the quality of life of postoperative patients [10, 11] Cassidy et al. [12] and Hall et al. [13] suggested that effective nursing measures can reduce the occurrence of related complications. However, what kind of nursing measures should be taken for patients after cardiac valve replacement to run through the postoperative rehabilitation process and improve patients’ medication compliance is the top priority in clinical nursing work. The phased written health education combined with healthy diet means that nursing staff will implement different content of health and diet education for patients at different stages after heart valve replacement. Its purpose is to improve the pertinence of health education and healthy diet, to provide patients with a comprehensive, scientific, targeted postoperative rehabilitation care. The results of this study showed that in the first-stage rehabilitation, the second-stage rehabilitation and the recovery period, the study group’s medication compliance was better than the control group (*P* < 0.05), suggesting that phased written health education combined with health diet can effectively improve patient compliance with medication. This may be due to the continuous strengthening of patients’ anticoagulation knowledge after heart valve replacement during the implementation of a phased written health education combined with healthy diet, so as to make patients fully aware of the necessity of taking medication and thus improve their medication compliance.

### Effect of phased written health education combined with healthy diet on anticoagulant drug knowledge in patients after heart valve replacement

Li Xue-ping et al. [14] suggested that the evaluation of the effect of continuous health education could effectively improve the knowledge of anticoagulant drugs in patients after heart valve replacement. Zeng Biru et al. [15] also confirmed that comprehensive nursing intervention can effectively improve the understanding rate of anticoagulation knowledge of patients after heart valve replacement. The above results fully demonstrated that effective nursing measures can effectively improve the rate of knowledge of anticoagulant drugs in patients after heart valve replacement, which is consistent with the results of our study. This is mainly because anticoagulant therapy is an effective measure to prevent thromboembolism in patients after heart valve replacement, phased written health education combined with health education in healthy diet is an important part of this nursing model. In the phased written health education process, the education team needed to tell the patient the role, effect, dosage and possible complications of anticoagulant drugs in an intuitive and repetitive manner. Also they should correctly identify possible complications and adopt effective preventive measures to encourage patients to fully grasp the relevant knowledge of anticoagulant drugs to reduce the occurrence of complications and ensure rational drug use and correct the possible complications.

### Effect of phased written health education combined with healthy diet on healthy behavior ability in patients after heart valve replacement

For patients after heart valve replacement, healthy behavior is very important. Such patients should maintain good living habits while insisting on using anticoagulant drugs, develop good healthy eating habits, and avoid using vitamin K-containing drugs.

The results of our study showed that the healthy behavior ability of the study group was better than that of the control group at all stages after surgery (*P* < 0.05), suggesting that the use of phased written health education combined with healthy diet can effectively improve patients’ healthy behavior ability. The phased written health education combined with healthy diet in this study not only formulated written health education content and healthy diet recommendations for patients, but also combined communication technology to communicate with patients through WeChat, established WeChat groups and WeChat public accounts. The team regularly sent health education content and dietary advice to patients through social software to ensure the immediacy of health education. Researches conducted by Wang Lei et al. [16] and Liu Xia et al. [17] also suggested that phased education could optimize the nursing process and correct the unhealthy behaviors of patients, the results were similar to the results of our study.

### The effect of phased written health education combined with healthy diet on the quality of life of patients after heart valve replacement

With the transformation of the medical model from a biological model to a bio-psycho-society-environmental comprehensive medical model, the quality of life has gradually been applied to the medical field, and has become one of the important indicators for evaluating patient treatment and prognosis. In patients who underwent heart valve replacement surgery, long-term preoperative poor heart function, surgical trauma, postoperative complications, and postoperative anticoagulant drug treatment could cause many health problems. Many factors could affect the quality of life. For example, the postoperative recovery of most patients is relatively slow due to advanced age, the original life pattern has changed, regular blood tests are required for anticoagulation therapy, and concerns about long-term related complications, etc., and the above factors are intertwined. He Jing et al. [18] suggested that the quality of life of patients with heart valve disease was significantly reduced after surgery, and effective symptom management is required. Luo Ji et al. [19] suggested that effective nursing interventions for patients discharged after heart valve replacement could improve the quality of life. Ge Yuting [20] also confirmed that effective nursing interventions could improve the quality of life of patients.

The results of our study were consistent with those of previous studies, suggesting that the combination of phased written health education combined with healthy diet can effectively improve the quality of life of patients after heart valve replacement.

In conclusion, the use of phased written health education combined with healthy diet can effectively improve patients’ medication compliance and anticoagulant knowledge at different stages, and at the same time encourage patients to improve their health behavior ability, thereby improving the quality of life, and it is worthy of clinical application. However, a larger sample size and longer time are needed to further clarify the assessment of the safety of patients after heart valve replacement by phased written health education and healthy diet.

## Data Availability

The datasets generated and analyzed during the current study are available from the corresponding author on reasonable request.
